# The Ancient Roman Circus

**Published:** 1885-12

**Authors:** 


### THE ANCIENT ROMAN CIRCUS.

In the earliest times this was nothing more than a flat, open space, round which temporary wooden platforms or scaffolds were raised for the spectators to stand upon ; but even before the destruction of the monarchy a permanent building was constructed for the purpose, and laid out upon a regular plan ever afterward retained until the final dissolution of the empire, and then the entire edifice, with its race-course and appendages, was included under the general name of circus. The ground plan was laid out in an oblong form, terminating in a semi-circle at one extremity, and inclosed at the opposite end by a pile of buildings called "the town," under which the stalls for the horses and chariots were distributed. A long, low wall was built lengthwise down the course, so as to form a barrier by which it was divided into distinct parts, and at each of its ends was placed a goal, round which the chariots turned, the one nearest the stable being called meta prima and the furtherest one meta secunda. As regards the external and internal elevation, the circus was constructed upon a similar design to that adopted for theatres and amphitheatres, consisting on the outside of one or more stories or arcades, according to the size and grandeur of the building, through which the spectators entered upon the staircase leading into the interior of the fabric The interior was arranged in rows of seats, divided into tiers, and separated by stairs and landing places.—*American Cultivator*.

